# Coronary artery calcification detected on low‐dose computed tomography in high‐risk participants of an Australian lung cancer screening program: A prospective observational study

**DOI:** 10.1111/resp.14832

**Published:** 2024-09-24

**Authors:** Asha Bonney, Michelle Chua, Mark W. McCusker, Diane Pascoe, Subodh B. Joshi, Daniel Steinfort, Henry Marshall, Jeremy D. Silver, Cheng Xie, Sally Yang, Jack Watson, Paul Fogarty, Emily Stone, Fraser Brims, Annette McWilliams, XinXin Hu, Christopher Rofe, Brad Milner, Stephen Lam, Kwun M. Fong, Renee Manser

**Affiliations:** ^1^ Department of Medicine University of Melbourne Melbourne Victoria Australia; ^2^ Department of Respiratory and Sleep Medicine Royal Melbourne Hospital Melbourne Victoria Australia; ^3^ Department of Radiology Royal Melbourne Hospital Melbourne Victoria Australia; ^4^ Department of Radiology University of Melbourne Melbourne Victoria Australia; ^5^ Department of Cardiology Royal Melbourne Hospital Melbourne Victoria Australia; ^6^ Thoracic Research Centre University of Queensland Chermside Queensland Australia; ^7^ Department of Thoracic Medicine The Prince Charles Hospital Chermside Queensland Australia; ^8^ Statistical Consulting Centre, School of Mathematics and Statistics University of Melbourne Melbourne Victoria Australia; ^9^ Respiratory Department Epworth Eastern Hospital Box Hill Victoria Australia; ^10^ Department of Thoracic Medicine and Lung Transplantation St Vincent's Hospital Darlinghurst New South Wales Australia; ^11^ University of New South Wales, School of Clinical Medicine, St Vincent's Clinical School; School of Population and Global Health University of Melbourne Melbourne Victoria Australia; ^12^ Department of Respiratory Medicine Sir Charles Gairdner Hospital Nedlands Western Australia Australia; ^13^ Curtin Medical School Curtin University Bentley Western Australia Australia; ^14^ National Centre for Asbestos Related Diseases Institute for Respiratory Health Nedlands Western Australia Australia; ^15^ Department of Respiratory Medicine Fiona Stanley Hospital Murdoch Western Australia Australia; ^16^ Department of Medicine University of Western Australia Nedlands Western Australia Australia; ^17^ Department of Medicine The University of British Columbia Vancouver British Columbia Canada

**Keywords:** cardiovascular disease, computed tomography, coronary artery calcification, lung cancer, screening

## Abstract

**Background and Objectives:**

Coronary artery calcification (CAC) is a frequent additional finding on lung cancer screening (LCS) low‐dose computed tomography (LDCT). Cardiovascular disease (CVD) is a major cause of death in LCS participants. We aimed to describe prevalence of incidental CAC detected on LDCT in LCS participants without prior history of coronary artery disease (CAD), evaluate their CVD risk and describe subsequent investigation and management.

**Methods:**

Prospective observational nested cohort study including all participants enrolled at a single Australian site of the International Lung Screen Trial. Baseline LDCTs were reviewed for CAC, and subsequent information collected regarding cardiovascular health. 5‐year CVD risk was calculated using the AusCVD risk calculator.

**Results:**

55% (226/408) of participants had CAC on LDCT and no prior history of CAD, including 23% with moderate–severe CAC. Mean age of participants with CAC was 65 years, 68% were male. 53% were currently smoking. Majority were high risk (51%) or intermediate risk (32%) of a cardiovascular event in 5 years. 21% of participants were re‐stratified to a higher CVD risk group when CAC detected on LCS was incorporated. Only 10% of participants with CAC received lifestyle advice (only 3% currently smoking received smoking cessation advice). 80% of participants at high‐risk did not meet guideline recommendations, with 47% of this group remaining without cholesterol lowering therapy.

**Conclusion:**

LCS with LDCT offers the potential to identify and communicate CVD risk in this population. This may improve health outcomes for high‐risk LCS participants and further personalize management once screening results are known.

## Abstract 2

Almost 25% of screening participants had moderate–severe CAC detected on LDCT and no known history of coronary artery disease. Most of these participants were at high risk of CVD and were not meeting guideline recommendations for CVD care. LDCT detected CAC may be a useful tool to optimize CVD health.

**See related** editorial

## INTRODUCTION

Lung cancer is the leading cause of cancer‐related death and screening with low‐dose computed tomography (LDCT) has been shown to reduce lung cancer‐related mortality by 21% and all‐cause mortality by 5% in high‐risk populations (people with a history of active tobacco smoking).^1^ The Australian Government has announced plans to implement a National Lung Cancer Screening Program (NLCSP) by mid‐2025.^2^

Coronary artery calcification (CAC) is a frequent incidental finding detected on LDCT performed for lung cancer screening (LCS), with the reported prevalence ranging from 21% to 94%.^3^ CAC correlates with atherosclerotic plaque burden and is a well‐established predictor of cardiovascular events.^4^ Not only was ischaemic heart disease the leading cause of death in Australia in 2022,^5^ cardiovascular disease (CVD) was also the leading cause of death in the largest LDCT LCS randomized controlled trial to date (National Lung Screen Trial; NLST).^6^ LCS with LDCT may be an opportunity to detect CVD early and intervene to reduce morbidity and mortality. Several measures have been evaluated to detect CAC on non‐cardiac gated LDCT, including simple visual assessment, ordinal scoring and a modified Agatston score.^6^, ^7^ These measures have been shown to be predictive of cardiovascular events in a LCS setting in people with a significant history of active tobacco smoking.^3^, ^6^ It should also be recognized that LCS populations tend to be higher risk for CVD based on age and active tobacco smoking histories, and 10% of cardiovascular events in the NLST occurred in people without CAC on LDCT.^6^

The recently published European joint statement on management of incidental findings from LCS with LDCT described the importance of reporting the presence and severity of CAC and highlighted a gap in our current knowledge surrounding the role of CAC detected on LDCT on existing risk stratification tools.^3^

The primary objectives of this study were to describe the prevalence and severity of CAC detected on LDCT in an Australian LCS cohort, its impact on CVD risk and describe subsequent investigations and management of incidental CAC in participants without a known history of coronary artery disease (CAD).

## METHODS

This prospective observational study was nested within a single Australian International Lung Screen Trial site (ILST; clinicaltrials.gov identifier: NCT02871856). The ILST protocol has previously been published.^8^ In Australia, the ILST recruited 2099 women and men aged 55–80 years old, with an active smoking history, and either an estimated 6‐year lung cancer risk of ≥1.51% based on the PLCOm2012 risk prediction model or ≥30 pack‐year smoking history, with a ECOG performance status of 0 or 1. ILST exclusion criteria are described in Table S1 in the Supporting Information.

Those enrolled in the study underwent a baseline LDCT to screen for lung cancer with further interval scans and investigation for lung cancer determined as per protocol.^8^ The LDCT (120 kV tube voltage, 40–50 mA tube current) was performed without intravenous contrast in the supine position. Scanning was performed during a single inspiratory breath hold. LDCT reporting was completed by experienced chest radiologists (≥300 CT chest readings in the last 3 years) using a standardized research template. A copy of the LDCT report was sent to participants' general practitioners (GPs), not directly to participants, and participants were informed to see their GP for results. No routine suggestions were provided to GPs regarding management of incidental CAC.

### Data collection

Age, sex, smoking status, smoking pack‐year history, ethnicity, co‐morbidities and medications were collected at baseline as part of the health questionnaire for all participants. At one ILST site, all participants with CAC on baseline LDCT were contacted via telephone to complete a once‐off CVD specific follow‐up questionnaire (included in Table S2 in the Supporting Information), focused on incidental findings, cardiovascular health and subsequent investigations, referrals, interventions and prescriptions, at variable timepoints from 18 months, up to 4 years post baseline LDCT. Confirmatory reports and records were also collected from treating clinicians and providers for all participants. This included blood pressure measurements, lipid profiles, renal function, blood glucose and/or glycated haemoglobin (HbA1c) and medication lists required to complete the cardiovascular risk assessment.

CAC was recorded as absent, mild, moderate or severe based on simple visual assessment on all screening LDCTs research reports,^6^ these results were then communicated to the participant's GP in the LDCT report. A second chest radiologist, blinded to the first assessment, rescored all baseline LDCTs identified with CAC using an ordinal scoring method as part of quality assurance for this study.^7^

### Outcomes

The primary outcomes of this study were to describe the prevalence of incidental CAC and to describe overall cardiovascular risk and subsequent investigations and management of incidental CAC in an Australian LCS population. We collected information on the proportion of participants who recalled being informed of incidental CAC detected on LDCT, and proportion of participants who received appropriate follow‐up as recommended, based on incidental CAC according to the Australian guideline for assessing and monitoring cardiovascular disease.^9^ Individual cardiovascular risk was assessed using the Australian AusCVD risk calculator which incorporates age, sex, smoking status, systolic BP, lipid profile, diabetes status, CVD medications (anti‐hypertensives, lipid modifying medication, anti‐platelet and anti‐coagulation medication) and history of atrial fibrillation.^10^ We chose to not include additional diabetes variables to calculate CVD risk as they were not routinely available for all participants. Low risk was defined as <5% chance of a cardiac event or stroke in the next 5 years, intermediate was 5 to <10% risk and high risk was ≥10% risk.

### Statistical analysis

Statistical analyses were performed in R.^11^ Statistics were predominantly descriptive, with continuous numeric data reported as means and standard deviations (SDs). Hypothesis tests for association between pairs of categorical variables were assessed using Fisher's exact test (for two dichotomous variables), Goodman–Kruskal's gamma statistic (for pairs of ordinal variables, or for one ordinal and one dichotomous variable) or Pearson's chi‐squared (for all other cases). A Goodman–Kruskal's gamma statistic score of 0 indicated an absence of association (range −1 to 1).^12^ Cohen's Kappa Coefficient was used to measure inter‐rater reliability. Kappa of <0 was less than chance agreement, 0.01–0.2 was slight agreement, 0.21–0.4 fair agreement, 0.41–0.6 moderate agreement, 0.61–0.8 substantial agreement and 0.81–0.99 almost perfect agreement.^13^ A *p*‐value of <0.05 was considered significant.

### Ethics approval

This study was approved by The Prince Charles Hospital Human Research Ethics Committee (HREC/16/QPCH/181) with all appropriate local governance approvals. All study participants provided written informed consent. The study was registered with clinicaltrials.gov (ID: NCT02871856).

## RESULTS

At one Victorian ILST site, 408 participants were enrolled. Almost all participants enrolled (99.5%) self‐referred to participate in ILST from advertising, with only two participants (0.5%) being referred to the ILST via their GP. Of those, 263 participants (64%) were identified as having CAC on LDCT. This Victorian site was representative of other Australian ILST sites, with 67% of Australian participants overall across the five sites having incidental CAC detected on baseline LDCT. Prevalence of incidental CAC on LDCT across other Australian sites is presented in Table S3 in the Supporting Information. Baseline demographics of our single site cohort with CAC are presented in Table 1. This study focused on the 226 participants with incidental CAC without a prior history of CAD. Follow‐up questionnaires were completed with 78% of these participants (177/226), in addition to collateral health care provider information. Eleven participants withdrew from the study and only baseline information was included, and the remaining 39 participants had follow‐up information collected only from treating clinicians and health care providers. There was no significant difference in age, sex and smoking status between those with and without a known history of CAD.

**TABLE 1:** Participant demographics by history of known coronary artery disease (CAD) with incidental CAC on LDCT.

	No known history CAD, *N* = 226	Known history of CAD, *N* = 37	*p*‐value^a^
Age, years	65 (6.1)	65 (6.7)	0.80
Sex			0.13
Female (%)	71 (31%)	7 (19%)	
Male (%)	155 (69%)	30 (81%)	
Smoking status			0.21
Current	120 (53%)	15 (41%)	
Former	106 (47%)	22 (59%)	
Ethnicity			
African	1 (0.4%)	0 (0%)	
Asian	6 (2.7%)	0 (0%)	
Caucasian	215 (95.1%)	37 (100%)	
Indigenous	2 (0.9%)	0 (0%)	
Other	2 (0.9%)	0 (0%)	
Co‐morbidities			
Hyperlipidemia	75 (33.2%)	21 (56.8%)	0.007
Diabetes	24 (10.6%)	3 (8.1%)	1.0
Stroke	8 (3.5%)	3 (8.1%)	0.19
Obesity	52 (23.0%)	16 (43.2%)	0.014

*Note*: For all variables except age, values shown are the number of participants (percentage of participants). For the Age variable, the mean (SD) is provided.

^a^ Test of statistical difference, Fisher's exact test for sex, smoking status and co‐morbidities, and a *t*‐test for differences between group means for age. Significant when *p* < 0.05.

Based on ordinal scoring, 17, 77,132 participants had severe, moderate and mild CAC respectively and no known history of CAD. There was moderate agreement between ordinal scoring and simple visual assessment (Cohen's Kappa of 0.41, 95% Confidence Interval (CI) 0.30, 0.53, *p*‐value <0.001). Scoring by simple visual assessment and ordinal scoring are presented in Table S4 in the Supporting Information. Fifty‐one participants (22%) had their CAC severity increased on re‐assessment with ordinal scoring. Sixteen participants' (7%) CAC severity decreased on re‐assessment, including one participant who as re‐assessed as having no CAC on LDCT. The majority of participants (160/227) did not change category and only 2 participants (0.9%) were reclassified by more than one category (2 with mild CAC on simple score were reclassified as severe on the ordinal score).

Ninety‐three percent (211/226 participants) had adequate information to calculate CVD risk using the AusCVD risk calculator. CVD risk of participants without a known history of CAD are presented based on AusCVD calculation alone and based on AusCVD calculation plus CAC severity (Table 2). The optional AusCVD postcode variable was omitted to avoid presumption and potential bias in evaluation the socio‐economic status of each participant. Eighty‐six participants with mild CAC (70% of participants with mild CAC and variables to complete CVD risk) were intermediate to high risk of CVD based on AusCVD calculation (Table S5). Forty‐five (21%) participants without known CAD were re‐stratified to a higher risk of CVD when incidental CAC was incorporated (Table 2). Of those where CAC resulted in a higher CVD risk category (10 participants changed from low risk to intermediate risk and 35 participants with intermediate risk changed to high risk), 44% (20/45) were on cholesterol lowering therapy at baseline. Seven participants in this group (16%) were on anti‐platelet therapy at baseline.

**TABLE 2:** CVD risk with and without CAC severity included.

		Risk of CVD (incorporating LDCT CAC severity)			
		Low	Intermediate	High	Total
Risk of CVD without CAC severity	Low risk	36	10^a^	0	46
	Intermediate	0	58	35^a^	93
	High	0	0	72	72
	Total	36	68	107	211

^a^ Participants who were re‐stratified to a higher CVD risk by incorporating CAC.

Most participants (172/226) reported no dyspnoea or chest pain at baseline.

Most participants were not on cholesterol lowering therapy or anti‐thrombotic therapy. Cholesterol lowering and anti‐thrombotic therapy prior to baseline LDCT are described in Table S6 in the Supporting Information by CVD risk and by CAC severity in Table S7 in the Supporting Information. Only 32% and 49% of people at intermediate and high CVD risk (not incorporating incidental CAC) respectively were on cholesterol lowering therapy at baseline. Only 65% of participants with severe incidental CAC were on cholesterol lowering therapy at baseline. Twenty‐two percent of people at high risk of CVD (not incorporating incidental CAC) were on anti‐platelet therapy and 4% were on anti‐coagulation.

Follow up of incidental CAC is described in Table 3. Although all participants were offered referral to quit services as part of the ILST, only 3% (3/90) of participants currently smoking with follow‐up data available received smoking cessation advice in the setting of CAC on LDCT.

**TABLE 3:** Follow‐up of incidental CAC in participants without a known history of CAD.

	# Responses^a^	Mild^b^	Moderate^b^	Severe^b^	Goodman–Kruskal's gamma estimate	Total # participants (% overall with known data)
Saw GP for results	174	80 (76%)	47 (82%)	9 (75%)	0.100	136 (78%)
Notified of incidental CAC	178	27 (25%)	16 (28%)	7 (54%)	0.180	50 (28%)
Additional investigations for CAC post screening	178	17 (16%)	8 (14%)	5 (39%)	0.095	30 (17%)
Medication changes for incidental CAC	178	8 (7%)	4 (7%)	5 (39%)	0.262	17 (10%)
Provided lifestyle advice (including smoking cessation)	174	6 (6%)	3 (5%)	1 (8%)	0.042	10 (6%)
Referred to cardiologist	178	13 (12%)	7 (12%)	6 (46%)	0.323	26 (15%)
Invasive testing (coronary angiogram)	174	3 (3%)	5 (9%)	3 (25%)	0.624	11 (6%)

^a^ Number of participants with a complete response to the outcome. Response was considered unknown (missing data) if unable to be confirmed with either participant or health care provider.

^b^ % of participants with known data.

There was no significant association between CVD risk category and meeting recommendations for care (Goodman–Kruskal's gamma estimate = −0.126, 95% CI −0.507, 0.154, *p*‐value = 0.39). Recommendations by CVD risk are presented in Table 4. Method of reporting CAC (highlighting CAC in the conclusion compared to including it only in the body of the report) demonstrated a moderate association with meeting recommendations (Goodman–Kruskal's gamma estimate of 0.584, 95% CI 0.336, 0.832 *p*‐value = 0.003). Reporting dyspnoea and/or chest pain was more frequent among those not meeting CVD risk recommendations (odds ratio = 0.431, 95% CI 0.183, 0.950, Fisher's exact test *p*‐value = 0.025). Less than half (16/45) of participants who had their CVD risk category increased by incorporating incidental CAC met recommendations for care.

**TABLE 4:** Outcomes by CVD risk (incorporating CAC).

	Risk of CVD (incorporating LDCT CAC severity)		
	Low	Intermediate	High
Met recommendations of care.	10 (27%)	15 (23%)	20 (20%)
Medications started after LDCT	4 (12%)	4 (7%)	10 (11%)
Cholesterol lowering	3 (8%)	3 (4%)	5 (5%)
Anti‐platelet	1 (3%)	2 (3%)	8 (8%)
Other	2 (5%)	1 (1%)	0 (0%)
Total # of participants on cholesterol lowering therapy after LDCT	14 (38%)	21 (31%)	56 (53%)
Total	37	68	106

There was no significant association between CVD risk (including CAC) and medication changes as described in Table 4 (Goodman–Kruskal's gamma estimate of 0.032, 95% CI −0.414, 0.477, *p*‐value = 0.89). Medications commenced after LDCT are presented in Table 4. Medication prescription by CAC severity is presented in Table S8 in the Supporting Information.

## DISCUSSION

This is the first study in Australia to comprehensively evaluate incidental CAC detected on LDCT performed for LCS in high‐risk participants aged 55–80 years old with an active tobacco smoking history and incorporate CVD risk assessment. CAC was a common incidental finding in our cohort, with 86% (226/263) of those with CAC detected on LDCT (55% of the total cohort) having no known history of CAD. This included 17 participants (4% of total cohort) with severe CAC. After screening, only 9% (4/46) not on cholesterol lowering therapy at baseline were commenced on lipid lowering treatment despite incidental moderate or severe CAC detected on LDCT. Almost half (47%) of participants with ≥10% risk of a cardiovascular event within 5 years were not on any cholesterol lowering medication. In the setting of eligibility for a LCS program, our cohort had a very high rate of current tobacco smoking, however few participants received smoking cessation support to address CVD risk.

### Significance of CAC on LDCT

The presence of CAC on non‐cardiac gated LDCT is a predictor of cardiovascular events.^7^ An American study of 13,644 patients reported that the effect of statin therapy to prevent a major adverse cardiovascular event was directly related to severity of CAC and was beneficial in even those with mild CAC (number needed to treat (NNT) = 100).^14^ The American Heart Association clinical practice guidelines favours statin therapy for anyone aged 40–75 years old with a borderline risk or any severity of CAC, particularly if over the age of 55.^15^

Of note, the presence of moderate and severe CAC detected on LDCT in this study did have a direct impact in re‐stratifying 21% of participants to a higher CVD risk category. This highlights the under recognized potential of incidental CAC assessment in lung cancer screening in clinical practice and requires further evaluation to determine if this further enhances cost‐effectiveness and/or clinical efficacy of LDCT screening. In addition, most participants who had their CVD risk increased by incorporating CAC were not on cholesterol lowering therapy at baseline, and most did not meet recommendations for CVD risk management.

Our understanding of the impact of incidental CAC in lung cancer screening is evolving. As highlighted in the NLST, early LDCT lung cancer screening trials did not consistently report the presence of CAC and may have had a higher threshold for significant or actionable CAC.^16^ The NLST reported only 6.7% of participants (1776/26455) who underwent at least one LDCT had significant CAC without evidence of prior coronary artery intervention. A potential contributor to the significantly higher rates of CAC reported in our study is the concurrent use of PLCOm2012 eligibility criteria. The PLCOm2012 eligible ILST population tended to be older and more co‐morbid compared to the ≥30 pack‐year smoking history eligible population.^17^ Our single centre also had a higher proportion of participants with current active tobacco use (55%), compared to the NLST LDCT arm (48%).^18^

International guidance for LCS recommends reporting severity of CAC as absent, mild, moderate or severe using simple visual assessment, ordinal scoring or modified Agatston method.^3^, ^6^, ^19^ The mention of CAC in the conclusion of the LDCT report was the only variable associated with an improvement, albeit modest, in management outcomes. LCS guidelines, including LUNG‐RADS, suggest the presence of CAC should be reported using a structured approach that includes management guidance such as primary care cardiovascular risk assessment.^3^, ^19^, ^20^ Another method suggested by the United Kingdom's SUMMIT LCS study was to recommend all GPs perform a CVD risk assessment for screening participants and omit communicating CAC score.^21^ However, a previous systematic review evaluating identification of CAC and pharmaceutical and lifestyle preventative measures demonstrated an increase in initiation and continuation of these therapies in those with CAC compared to no CAC.^22^ This highlights the potential for CAC to be used as motivational strategy when engaging people in CVD risk management. Most of our cohort with mild CAC (73%) were still intermediate to high risk for cardiovascular events based on other variables.

### CVD health in LCS

This study demonstrates a major opportunity to improve health outcomes for an already high‐risk cohort of individuals. More than half of our cohort of LCS participants with incidental CAC were at high risk (≥10%) of a cardiovascular event in the next 5 years, with an additional 32% at intermediate risk (5 to <10%) in the next 5 years. However, most participants were unaware of the finding of incidental CAC on screening LDCT, and 79% of participants with incidental CAC and no known history of CAD did not have recommendations for CVD risk met. Most patients did not have any additional investigations performed, and very few received lifestyle advice or tobacco cessation treatment and medication changes. The potential to utilize incidental CAC detected on non‐cardiac CT to optimize cardiovascular health is underrecognized in Australia. An Australian study of 123 hospital patients who had non‐cardiac gated CT chest demonstrating CAC as an inpatient found that only 27% of CAC had severity reported and only 20% of patients had further investigations performed for CVD.^23^

### Future directions

Addressing physical activity levels, diet and alcohol consumption, smoking cessation and weight management have established roles in improving cardiovascular health in all risk categories.^9^ Recently Venkataraman et al. showed incorporating CAC into statin therapy decision making may be a more cost‐effective approach compared to adjusting CVD risk thresholds for treatment in people with a family history of premature CAD.^24^

Other international LCS studies have described undertreatment of CAC detected on LDCT.^25^, ^26^ In comparison with these studies a strength of our study was the availability of clinical information including medical history, lipid profiles and blood pressure measurements to accurately complete CVD risk assessment.

It is important as we implement LCS in Australia and other countries, that we consider evaluating the impact of LCS on cardiovascular outcomes and potential cost effectiveness of incorporating incidental CAC on LDCT into CVD risk assessment.

### Limitations

Our study was limited as a single centre with a smaller sample size of screening participants, although this site was representative of Australian ILST sites. Our population was predominantly Caucasian and had very few First Nations participants. Although objective CAC assessment is possible on non‐gated studies, this method is more time consuming and requires specialized software. As such we used previously described visual methods of assessment which are more likely to reflect clinical practice. Whilst long‐term follow‐up is still underway for the ILST, we did not have data available regarding cardiovascular outcomes for participants from national databases. Follow‐up information was limited to patient‐reported events after LDCT and clinician records and investigations. This sub study did not incorporate smoking cessation rates, although as part of the ILST, all participants with current active tobacco use were advised to quit and offered referral to quit line services. In one large American cohort study with 551,388 adults, 34.7% of cardiac deaths were attributed to smoking (RR 1.53, 95% CI 1.48, 1.59).^27^ As participants were able to self‐refer to the ILST, GPs were not involved in ordering the LDCT, and potentially there was less engagement with results. This is a significant practical consideration for LCS programs.

## CONCLUSION

This study highlights several areas for urgent consideration for Australia implementing a NLCSP in 2025, in addition to other countries planning to commence LCS. LCS participants, based on current age and tobacco smoking criteria, are at higher risk of CVD disease. Over a fifth of participants had their CVD risk estimate upgraded by incorporating incidental CAC detected as part of LCS. At present, most participants are not receiving standard of care to optimize their CVD risk including ongoing smoking cessation support. LCS provides a valuable opportunity to prompt CVD risk assessment and findings of incidental CAC should be communicated effectively to treating clinicians and participants with appropriate guidance on further management recommendations.

## AUTHOR CONTRIBUTIONS

**Asha Bonney:** Conceptualization (equal); data curation (lead); formal analysis (equal); funding acquisition (equal); methodology (equal); project administration (lead); resources (equal); visualization (equal); writing – original draft (lead); writing – review and editing (lead). **Michelle Chua:** Data curation (equal); methodology (equal); resources (equal); writing – review and editing (equal). **Mark W. McCusker:** Conceptualization (equal); data curation (equal); methodology (equal); resources (equal); writing – review and editing (equal). **Diane Pascoe:** Data curation (equal); methodology (equal); resources (equal); writing – review and editing (equal). **Subodh B. Joshi:** Methodology (equal); supervision (equal); writing – review and editing (equal). **Daniel Steinfort:** Supervision (equal); writing – review and editing (equal). **Henry Marshall:** Conceptualization (equal); project administration (equal); supervision (equal); writing – review and editing (equal). **Jeremy D. Silver:** Formal analysis (equal); resources (supporting); writing – review and editing (equal). **Cheng Xie:** Data curation (supporting); writing – review and editing (equal). **Sally Yang:** Data curation (supporting); writing – review and editing (equal). **Jack Watson:** Data curation (supporting); writing – review and editing (equal). **Paul Fogarty:** Data curation (supporting); writing – review and editing (equal). **Emily Stone:** Data curation (supporting); writing – review and editing (equal). **Fraser Brims:** Data curation (supporting); writing – review and editing (equal). **Annette McWilliams:** Data curation (supporting); writing – review and editing (equal). **XinXin Hu:** Data curation (supporting); writing – review and editing (equal). **Christopher Rofe:** Data curation (supporting); writing – review and editing (equal). **Brad Milner:** Data curation (supporting); writing – review and editing (equal). **Stephen Lam:** Conceptualization (equal); data curation (supporting); resources (equal); writing – review and editing (equal). **Kwun M. Fong:** Conceptualization (equal); data curation (supporting); funding acquisition (equal); methodology (equal); resources (equal); supervision (equal); writing – review and editing (equal). **Renee Manser:** Conceptualization (equal); methodology (equal); supervision (lead); writing – review and editing (equal).

## CONFLICT OF INTEREST STATEMENT

Daniel Steinfort and Emily Stone were on the Editorial Board of Respirology at the time of review. They were excluded from all editorial decision‐making related to the acceptance of this article for publication. All remaining authors have no competing interests to disclose.

## HUMAN ETHICS APPROVAL DECLARATION

This study was approved by The Prince Charles Hospital Human Research Ethics Committee (HREC/16/QPCH/181) with all appropriate local governance approvals. All study participants provided written informed consent. The study was registered with clinicaltrials.gov (ID: NCT02871856).

## Supporting information


**Data S1:** Supporting Information.

## Data Availability

The study data can be accessed by contacting the corresponding author.
