# Supplementary material for: Coronary artery calcification detected on low‐dose computed tomography in high‐risk participants of an Australian lung cancer screening program: A prospective observational study
Source: Respirology. 2024 Sep 24;30(1):62–9. doi: 10.1111/resp.14832 (PMC11688628; doi:10.1111/resp.14832)
Supplement: Supplementary file 1 — Data S1: Supporting Information. [file RESP-30-62-s001.docx]

Supplementary Materials

**Coronary artery calcification detected on low-dose computed tomography in high-risk participants of an Australian lung cancer screening program: a prospective observational study.**

***Table S1****. ILST Exclusion Criteria*

| - Clinical symptoms suspicious for lung cancer. - Any medical condition that could jeopardise safety to participate or ability to benefit from screening. - Previous diagnosis of lung cancer. - Previous non-curative treatment of cancer outside the lung or less than 5 years cancer free. - Pregnancy. - Pneumonia within the last 12 weeks. - CT chest performed within the last 2 years. - Unable to lie in the position for the CT. - Received cytotoxic drugs within the last 6 months. - Unwilling to sign consent. |
| --- |

***Table S2****. Summary of questions contained in the cardiovascular disease telephone follow-up questionnaire.*

| 1. Are you aware of any personal history of heart disease?    1. When was this diagnosed?    2. Where was this diagnosed?    3. What treatment are you currently on?    4. Who do you see for management of your heart disease? 2. Do you have    1. High blood pressure?       1. Have you had any recent checks of your blood pressure?       2. What was the most recent reading?       3. Where was your last reading performed?    2. High cholesterol?       1. Have you had any checks for your cholesterol levels?       2. Where and when were these performed?    3. Irregular heart rhythms?       1. Have you had an electrocardiogram (ECG) or trace of your heart performed?       2. If so, where and when?    4. Diabetes?       1. Have you had any checks of your blood sugar levels?       2. Where and when were they performed? 3. Can I confirm your current medications?    1. Are you on a statin or cholesterol medication?       1. When did you start this treatment (before or after screening)?    2. Are you on aspirin or any blood thinning medications?       1. When did you start this treatment (before or after screening)?    3. What is your current smoking status?    4. Do you experience any chest pain/discomfort or breathlessness?       1. Have you seen a doctor about this? *If no, recommend consultation* 4. Do you have any family history of heart disease? 5. Do you still have a regular family/GP doctor that you see?    1. Who is your current GP? 6. Did your doctor inform you of any “incidental” or unexpected findings from your trial CT scan?    1. What were they?    2. Were you aware that the screening may detect other findings besides lung cancer?    3. *If informed of cardiac disease*       1. What is your understanding of [heart disease or coronary artery calcification]? 7. Did you have any additional investigations following your CT?    1. When and where were they performed? 8. Did you have any changes in your medications as a result of the CT scan or subsequent investigations?    1. What were they? 9. Did you have any other changes to your care, for instance lifestyle advice regarding diet and exercise?    1. What were they?    2. Did you follow the advice? 10. Did you have any subsequent referrals to see another clinician (such as a cardiologist, physiotherapist, dietician) or clinic?     1. Who were they?     2. What were the recommendations?     3. Did you follow the recommendations? 11. Did you have any invasive procedures performed as a result of your CT scan?     1. What were they and when were they performed? 12. The next few questions are in regards to your relationship with your GP and your experience of the communication.     1. Did you have a discussion/appointment with your GP regarding the results of the first scan?     2. On the following scale (1=Poor, 2=Fair, 3=Good, 4=Very good, 5=Excellent, 6=Not applicable, 7= don’t know), how would you rate each statement?        1. Your GP explained the results of your CT scan.        2. Your GP involved you in the decisions.        3. Your GP had enough time to listen to what you had to say.        4. Your GP allowed you to have the final choice about decisions made.        5. Your GP gave you information on how to prevent future health problems.        6. Your GP arranged treatment for you.        7. You are confident in the care with your GP provides. |
| --- |

***Table S3****. Incidental CAC detected on baseline LDCT by site in Australia.*

|  | New South Wales | Queensland | Victoria 1 | Victoria 2 | Western Australia |
| --- | --- | --- | --- | --- | --- |
| Total number of participants | 378 | 595 | 408 | 127 | 591 |
| Number with incidental CAC (%) | 204 (54%) | 419 (71%) | 263 (64%) | 79 (66%) | 432 (74%) |

***Table S4****. CAC severity by simple visual assessment and ordinal scoring.*

|  |  | CAC (ordinal) | | | | |
| --- | --- | --- | --- | --- | --- | --- |
|  |  | **Absent** | **Mild** | **Moderate** | **Severe** | **Total** |
| **CAC (simple visual assessment)** | **Absent** | 0 | 2 | 0 | 0 | 2 |
|  | **Mild** | 1 | 115 | 39 | 2 | 157 |
|  | **Moderate** | 0 | 15 | 38 | 8 | 61 |
|  | **Severe** | 0 | 0 | 0 | 7 | 7 |
|  | **Total** | 1 | 132 | 77 | 17 | 227 |

***Table S5****. Baseline CVD risk (not incorporating CAC) by incidental CAC severity.*

|  | Mild | Moderate | Severe | Total |
| --- | --- | --- | --- | --- |
| Low risk | 37 | 8 | 2 | 47 |
| Intermediate risk | 57 | 36 | 2 | 95 |
| High risk | 28 | 28 | 12 | 68 |
| Risk unknown | 10 | 5 | 1 | 16 |
| Total | 132 | 77 | 17 | 226 |

***Table S6****. Number of participants with incidental CAC without known CAD prescribed cholesterol lowering and anti-thrombotic therapy at pre-screening.*

|  | Risk of CVD (without CAC severity) | | |
| --- | --- | --- | --- |
|  | **Low** | **Intermediate** | **High** |
| **Cholesterol lowering therapy*** | 15 (33%) | 30 (32%) | 35 (49%) |
| **Anti-platelet therapy**** | 3 (7%) | 10 (11%) | 16 (22%) |
| **Anti-coagulation***** | 0 (0%) | 0 (0%) | 3 (4%) |
| **Total participants** | 46 | 93 | 72 |

*There was an association between CVD risk at baseline and being on cholesterol lowering therapy (Goodman-Kruskal’s gamma = 0.237, 95% CI 0.010, 0.463, p-value 0.04).

** There was an association between higher CVD risk and being on anti-platelet medication (Goodman-Kruskal’s gamma = 0.430, 95% CI 0.136, 0.725, p-value =0.010).

***There was no significant association between CAC severity on LDCT and being on anti-coagulation (Pearson’s Chi^2^ p-value = 0.052).

***Table S7****. Number of participants within incidental CAC without known CAD prescribed cholesterol lowering and anti-thrombotic therapy at baseline.*

|  | CAC Severity | | |
| --- | --- | --- | --- |
|  | **Mild** | **Moderate** | **Severe** |
| **Cholesterol lowering therapy*** | | | |
| Yes | 35 (27%) | 36 (47%) | 11 (65%) |
| No | 96 (73%) | 41 (53%) | 5 (29%) |
| **Anti-platelet therapy**** | | | |
| Yes | 15 (11%) | 15 (19%) | 1 (12%) |
| No | 115 (87%) | 62 (81%) | 15 (88%) |
| **Anti-coagulation***** | | | |
| Yes | 2 (2%) | 1 (1%) | 1 (12%) |
| No | 129 (97%) | 76 (99%) | 15 (88%) |
| **Unknown** | 1 (1%) | 0 (0%) | 1 (6%) |
| **Total participants** | 132 | 77 | 17 |

*There was an association between CAC severity on LDCT and being on cholesterol lowering therapy (Goodman-Kruskal’s gamma = 0.476, 95% CI 0.276, 0.676, p-value<0.001).

**There was no association between CAC severity on LDCT and being on anti-platelet therapy (Goodman-Kruskal’s gamma = 0.171, 95% CI -0.148, 0.490, p-value=0.318).

***There was no significant association between CAC severity on LDCT and being on anti-coagulation therapy (Goodman-Kruskal’s gamma = 0.264, 95% CI -0.602, 1.129, p-value=0.600).

***Table S8****. Number of participants with medications commenced post LDCT by CAC severity.*

|  | CAC Severity | | |
| --- | --- | --- | --- |
|  | **Mild** | **Moderate** | **Severe** |
| **Cholesterol lowering** | 7 | 2 | 2 |
| **Anti-platelet** | 3 | 4 | 4 |
| **Glyceryl trinitrate** | 1 | 0 | 0 |
| **Anti-hypertensive** | 2 | 0 | 0 |
